# New Target for Accurate Terrestrial Laser Scanning and Unmanned Aerial Vehicle Point Cloud Registration

**DOI:** 10.3390/s19143179

**Published:** 2019-07-19

**Authors:** Tilen Urbančič, Žiga Roškar, Mojca Kosmatin Fras, Dejan Grigillo

**Affiliations:** 1 Faculty of Civil and Geodetic Engineering, University of Ljubljana, Jamova cesta 2, 1000 Ljubljana, Slovenia; 2 Leica Geosystems AG, Heinrich-Wild-Strasse, CH-9435 Heerbrugg, Switzerland

**Keywords:** artificial target, terrestrial laser scanning, SfM photogrammetry, registration, registration quality

## Abstract

The main goal of our research was to design and implement an innovative target that would be suitable for accurately registering point clouds produced from unmanned aerial vehicle (UAV) images and terrestrial laser scans. Our new target is composed of three perpendicular planes that combine the properties of plane and volume targets. The new target enables the precise determination of reference target points in aerial and terrestrial point clouds. Different types of commonly used plane and volume targets as well as the new target were placed in an established test area in order to evaluate their performance. The targets were scanned from multiple scanner stations and surveyed with an unmanned aerial vehicle DJI Phantom 4 PRO at three different altitudes (20, 40, and 75 m). The reference data were measured with a Leica Nova MS50 MultiStation. Several registrations were performed, each time with a different target. The quality of these registrations was assessed on the check points. The results showed that the new target yielded the best results in all cases, which confirmed our initial expectations. The proposed new target is innovative and not difficult to create and use.

## 1. Introduction

Three-dimensional point clouds can be achieved through either active or passive remote sensing systems [1]. Active sensors, such as laser scanners, use the laser light to directly provide a precise and dense point cloud, which is properly scaled. Passive sensors, such as digital frame cameras, use feature matching in overlapping imagery (i.e., the automated identification of conjugate features in the involved images) to derive 3D information. With known interior and exterior orientation of the camera, a 3D point cloud can be computed with the intersection of image rays that belong to the image coordinates of conjugate points [2]. Interior orientation parameters can be obtained with camera calibration. Exterior orientation parameters can be derived through either a direct or indirect georeferencing procedure. A thorough review of image-matching algorithms is given in Reference [3]. The computer vision community introduced an approach for creating point clouds from overlapping images, known as structure from motion (SfM). In SfM, features (key points) are obtained from images using feature detectors and are matched to their descriptors. Different feature detectors, descriptors, and their combinations were tested in References [4,5,6]. In the next step, a sparse bundle adjustment is performed in order to calculate the camera parameters and transform image coordinates into 3D points. The sparse point cloud is then intensified using dense image-matching algorithms [7,8]. Structure from motion simultaneously estimates the exterior orientation parameters of the involved images and derives the 3D coordinates of corresponding features in those images. In the absence of control information, SfM-based point clouds are usually referred to an arbitrary reference frame [1]. The transformation of the point cloud in the reference coordinate system can be achieved by the use of ground control points (GCPs). These must be included as constraints in bundle adjustment rather than by just applying a 3D similarity transformation from an arbitrary reference system after SfM [9]. The SfM method has a large computational cost associated with image matching, and many efforts have been undertaken to reduce that cost. For example, a skeletal camera network was embedded into an SfM to enable efficient 3D scene reconstruction from a large set of unmanned aerial vehicle (UAV) images [10].

### 1.1. Overview of Point Cloud Registration Methods

To register point clouds acquired from different scenes, there are two main phases: The initial (coarse) alignment and the fine alignment [11,12,13]. Coarse alignment is best achieved by matching conjugate 3D features that have been extracted from two datasets [1,14]. Conjugate features could be points, lines, or planar features. The pairs of matched features are used to calculate the rigid body transformation from the “source” to the “target” point cloud. In cases where the scales of the registered point clouds are not uniform, a 3D similarity transformation is more appropriate [15]. In the next step, the registration process is refined using the fine alignment method. The most widely used fine registration method is the iterative closest point (ICP) algorithm [16] and its variants. The iterative closest point optimizes the transformation between two point clouds by minimizing the square errors between the correspondences. In its basic form, the number of points is selected from the point set to be registered, and the points corresponding to these points in the reference point set are then identified. The transformation is obtained by minimizing the distances between these pairs of points. Because of the irregular nature of point clouds, point-to-point correspondence cannot always be assumed [1]. Different variants of the ICP have been introduced for such situations. Improved methods based on ICP were reviewed in Reference [12]. Due to their automatic registration procedure, the variants of ICP algorithms are commonly used for registering point clouds [17,18,19,20,21,22,23,24,25].

The iterative closest point assumes a good estimation of the initial location of the point clouds to be registered. Therefore, the coarse registration already needs to provide a fairly accurate alignment of the two datasets. The complexity of coarse registering two datasets lies in the robust identification of conjugate features that must be spread as much as possible across the area. Feature extraction and a matching step can be approached by manual point-based matching, target-based matching, or feature-based matching [14]. The manual method requires the user to manually select matching points in both point clouds. This approach is not always reliable, mainly because of difficulties in selecting appropriate matching points. The target-based method aims at inserting objects (targets) into the scene, which can be easily recognizable and matchable in different point clouds. The feature-based method aims at automatically extracting and reliably matching 3D features from different datasets. According to the kind of feature used for registration, the methods can be put into three categories: Point-, line-, and plane-based methods [26].

### 1.2. Target-Based Registration

Point-based registration could be using point targets, individual points within the data, or key points that have characteristic attributes [1]. Target-based registration uses artificial targets, which are set up within the field of view of the data acquisition systems and are designed to facilitate their automatic identification from the derived point clouds.

Artificial targets are often used in registration procedures or when georeferencing a point cloud [27]. When deciding upon the suitability of an individual target, we need to consider its stability, rigidity, and surface reflectivity [28,29]. For targets that are used in laser scanning, it is characteristic that, in addition to the geometry of points, point intensity information is also used to determine the position of the target in the point cloud. The strongest signal should be returned from the center of the reflective target [30]. Targets are made from different materials. Retroreflective materials can be easily recognized even if very long distances are considered. Retroreflective targets reflect the incident beam along the same direction [31]. The varying reflectivity of materials causes different intensity values [32]. Target reflection properties also cause an offset in distance measurements [33,34]. Experimental results, such as those presented in Reference [31], have revealed the presence of large systematic range errors when scanning retroreflective target surfaces. A precise target-based positioning can only be achieved after modeling the systematic errors based on carefully designed calibration tests. The color of the targets is also important, because different colors produce different reflected intensities. A weighted total least squares method for precise black and white target measurement was proposed in Reference [35]. Artificial targets differ in shape, and geometrically they can be divided into plane and volume targets. Plane targets are more diverse, and certain equipment manufacturers have supplied us with a few characteristic ones. In addition, targets that are neither retroreflective nor made from special materials can be used for point cloud registration if they have a definite template shape [36]. Volume targets are designed in simple geometric forms, which are easy to model, such as spheres, cylinders, and cones. The use of volume targets for registering point clouds was studied and written about in References [37,38,39,40,41]. Rabbani and van den Heuvel [42] used planes, cylinders, and spheres as registration primitives. Their methodology of finding possible conjugate features was complemented by geometric constraints. Standard deviations when registering point clouds using their method were lower than those from ICP. The size and shape of the target should correspond to the maximum distance from the scanner, thus minimizing systematic errors due to the increase in laser spot size and assuring a high-density sampling of the surface [43]. The most important characteristic of the target is that it enables the simple modeling of its form, which in turn gives us precise coordinates of the reference target point in the various point clouds that we wish to merge. A reference target point is a geometrically defined point that can be uniquely defined in a point cloud, and in the case of plane targets also on images (center of a circle or a sphere, apex of a cone, etc.).

### 1.3. Feature-Based Registration

Linear and planar features have been repeatedly used as registration primitives for the coarse alignment of point clouds [1]. Researchers use those 3D features exclusively or in combinations to register point clouds. A methodology for registering photogrammetric datasets and light detection and ranging (LiDAR) data using 3D straight-line features instead of points was reported in Reference [15]. In Reference [44], line features were detected in both terrestrial and aerial laser scanner point clouds of built-up areas. The detected features were then used for registration in separate rotation and translation steps without any prior knowledge. The solutions were not stable, as the building interiors and exteriors could not be distinguished from line representation. In Reference [45], spatial curves were used as matching primitives to calculate the initial transformation parameters between the scanned point clouds of freeform surfaces (e.g., statues, cultural heritage artifacts). Al-Durgham and Habib [18] proposed an association matrix-based sample consensus approach for the registration of terrestrial laser scans using linear features. Their approach produced better results in fewer trials compared to the traditional random sample consensus (RANSAC) algorithm. A closed-form procedure for the coarse registration of 3D point clouds using automatically extracted linear features was presented in Reference [1]. The proposed method was capable of solving the registration problem in the presence of noisy measurements and providing reliable coarse alignment for a successful fine registration. The main limitation of the proposed approach was the reliance on manual identification of corresponding linear features in the point clouds. Bosché [14] automatically extracted planes to register terrestrial laser scanner (TLS) point clouds with industrial project 3D models. He calculated rigid transformation using two matches of nonparallel vertical planes and one match of horizontal planes. Three nonparallel planes may not always be present in construction scans, in which case the proposed approach fails. An alternative “2 planes + 1 point” registration approach was proposed in those cases. In Reference [46], terrestrial laser scanner (TLS) point clouds were registered with the method, which is based on virtual tie points that are generated by intersecting triples of scene planes. Planes in the point clouds were detected using RANSAC, which is often used to segment the planes in the point cloud. Other methods are also in use (e.g., a robust principal components analysis to segment linear and planar structures in point clouds was presented in Reference [47]). Xu et al. [48] reported an automatic marker-free method for fast and coarse registration between point clouds using the geometric constraints of planar patches, combining the voxel structure and a RANSAC-based strategy for selecting the corresponding plane pairs. The voxel structure suppressed the negative effects of outliers and unevenly distributed densities of points. In addition, using voxels instead of points to represent the data largely improved the efficiency of processing. Based on the voxel structure, a geometric constraint using small planar patches was designed to rapidly estimate the local coordinate frame of a scan in an urban scene. Compared to conventional ways of extracting planar surfaces with model fitting and region growing, the proposed planar constraint was generated in a more efficient way with a lower computational cost. In Reference [49], planar features were detected within unregistered scans. By registering planes instead of points, they reduced the amount of data that need to be processed by the hardware, and they successfully registered several thousand laser scans. Several researchers benefited by using combinations of extracted features to register point clouds. The least squares approach, which matches arbitrarily oriented 3D surface patches and curves, was presented in Reference [50]. In Reference [51], LiDAR point clouds were registered by employing point, line, and plane features. Depending on the scene geometry, each one of the features can be exclusively used or the features can be combined for calculating the transformation parameters. Due to combined measurements, the feature-based transformation model can afford a higher degree of flexibility and offer higher accuracy of point cloud registration tasks. Experimental results from this work showed that linear and planar primitives yield reliable estimates of the transformation parameters. Yao et al. [17] developed a RANSAC-based algorithm to register laser scans in a pairwise fashion, in which groups of linear and planar features are used as the registration primitives. They reported that the accuracy, robustness, and matching effectiveness of their registration method using planar features were better than with linear features. However, the algorithm was sensitive to the presence of repetitive patterns, and the registration of outdoor scenes usually failed. A non-iterative solution to estimate rigid transformation parameters using vector geometry from hybrid geometric features (including points, lines, and planes) was proposed in Reference [52]. The method does not require an initial alignment, and the estimated transformation parameters are at the same level of quality when using the classic least squares approach, but with an improved computational performance in real-field applications. A fully automatic registration procedure between mobile mapping and nadir airborne imagery to improve the localization quality of mobile mapping platforms in GNSS-denied areas was proposed in Reference [53]. They used common and clearly distinguishable elements, such as road markings, manholes, or curbstones, as conjugate features. The same authors expanded their procedures by including oblique imagery for the registration of both datasets [54]. They extracted façades in sparse point clouds created from terrestrial image correspondence and then used them as projection surfaces for both image datasets. The registration was conducted by a template matching approach. Besides geometry, semantic information about 3D features can contribute to the efficiency of point cloud registration. The registration of terrestrial point clouds, which uses semantic information to automate geometric feature matching, was proposed in Reference [55]. The semantic-aided feature matching concept leads to a more robust and efficient registration process. Yang et al. [26] proposed a marker-free and multiview registration method for large-scale urban scene point clouds based on semantic feature points. The main contribution of the proposed method was the detection of a small set of semantic feature points, matching them using both geometrical constraints (three-point scheme) and their semantic information (category and direction). The proposed method worked well even in cases where the overlap was only 15% between neighboring scans.

Besides providing the initial parameters for fine registration, coarse alignment is also important when there is not enough overlap between the registered point clouds. In fine registration, the main objective is to achieve the maximum overlap of two point clouds [12]. Even feature-based methods require overlap between point clouds: For example, at least three pairs of conjugate surfaces should be present in point clouds to be registered [26]. Problems with registration can occur when using multitemporal 3D point clouds obtained from data acquired at different times. The surface may change over time, and the point clouds might not be comparable. To overcome this problem, a multisensor and multidata integration approach performed on a common primary data level was presented in Reference [56]. Targets can be the appropriate primitives for the registration of datasets that are acquired at different times only if one can guarantee that the targets are set up at the same locations in all surveys [1]. An automatic technique to register multitemporal datasets from UAVs was presented in Reference [57]. The authors proposed a reference image block coregistration algorithm that uses reference anchor images to constrain the orientation of images acquired at different times. In order to produce good results for the registration, the anchor images must be well distributed and not include the changing area.

### 1.4. A New Target for TLS and UAV Image Point Cloud Registration

In this article, we focus on the registration of point clouds measured with TLS and produced from photogrammetric images taken from UAVs. Two different approaches can be adopted for the registration of 2D image-based and 3D laser point clouds [1]. The first approach is based on a 2D-to-3D alignment strategy, in which registration is achieved using identified features in the 2D images and 3D point cloud in question [58]. The second approach is based on a 3D-to-3D registration strategy. More specifically, a point cloud can be generated from 2D imagery through the automated identification of conjugate points in overlapping imagery. Then, the registration problem is solved through the alignment of image-based and laser scan point clouds [1].

Many examples that combine TLS and UAV image point clouds can be found in the scientific literature [11,13,19,21,23,24,59,60,61,62,63]. By reviewing that literature, we discovered that only a few studies have used special targets to register point clouds. To our knowledge, researchers have mostly relied on feature-based methods or fine registration algorithms or have registered point clouds merely on GCPs that were signalized by targets that were separated for both technologies. If common targets are used for TLS and UAV point clouds, their shape is planar.

Our aim was to design a target that would be appropriate for engineering tasks in smaller areas, where we cannot achieve high overlap between point clouds or there are not enough objects to successfully perform feature-based or fine-registration methods. The positional accuracy of TLS is usually much higher than that of SfM [23], and the resolution of the registered point clouds can vary. Therefore, the target should enable us to precisely measure the reference target point in TLS and UAV image datasets. In an innovative approach, we constructed a unique target that combines the properties of plane targets, which are usually used for the signalization of GCPs in photogrammetric images, and volume targets, which are often used to register laser scans. The orientation of planes whose normal vectors cover all main directions in space contributes to the successful registration of point clouds [55,64]. Hence, the “new target” (as we name it in this article) is composed of three perpendicular planes. Thus, we can describe it as a “three-plane target”. In an extensive experimental study, we compared and analyzed the quality of aerial and terrestrial point clouds that were registered using different commonly used plane and volume targets as well as the new target, hoping to establish whether the new target can yield better results.

## 2. Materials and Methods

### 2.1. New Target

**Figure 1:**
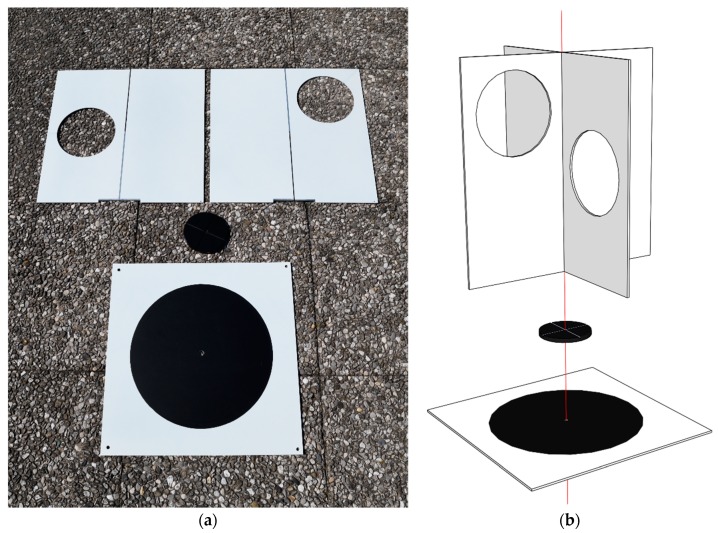
New target: (**a**) Target components; (**b**) upper part of the target, which is used for terrestrial laser scanning (TLS), and lower part of the target, which is used for unmanned aerial vehicle (UAV) surveys, are assembled with the connecting element.

In our study, we conceived and created a target that can be used for georeferencing and registering point clouds that are produced from UAV images and TLS point clouds. It comprises two parts: A normal UAV target (a black circle on a white background) and an additional upper part, which is used for TLS (Figure 1a). This target enables the determination of the reference target point in both datasets. The target is composed of 3 square aluminum panels, each measuring 40 cm × 40 cm × 0.2 cm, and a connecting element in the form of a discus with a diameter of 10 cm and a width of 0.8 cm. All panels are painted matte white, as this provides good reflection with its coarseness and shades.

In the event of photogrammetric surveying with a UAV, only the lower part of the target is used, i.e., the square panel, with a circle 30 cm in diameter marked on it with black matte vinyl foil. The reference target point is defined by the center of the circle.

When using TLS, the upper part of the target is attached. The rotating connecting element is attached to the UAV target, and two vertical, interconnected rectangular panels are attached to the connecting element (Figure 1b). The reference target point is defined by the cross-section of all three panels, which physically coincides with the center of the black circle. The alignment of the cross-section of the vertical panels with the center of the black circle on the lower part of the target is ensured by the connecting element, which has a rotating axis on the lower plane. This axis is attached to the hole in the center of the black circle in the horizontal panel. The upper plane of the connecting element has two rectangular slots that serve to stabilize and centralize the vertical panels. The vertical panels have circles with diameters of 15 cm cut into them, which enables us to measure the points on all four sections of the vertical panels while scanning and thus provides us with a better definition of the planes. The rotating connecting element enables us to turn the vertical panels while scanning so that the holes are turned toward the scanner. This improves the quality of the scanned points on the target, because the incidence angles on both vertical panels are lower and almost identical.

The dimensions of the target are primarily determined by the size of the black circle, which is often used for signalizing GCPs in photogrammetric UAV surveys. The circle diameter of 30 cm ensures that the target will be imaged with a sufficient number of pixels to perform automatic image measurements for the surveys at up to 100 m of altitude (depending on the camera’s focal distance). We limited the dimensions of the holes in the vertical plates to 15 cm in order to ensure the robust construction of the target. We scanned the target in different positions from the 10-m distance with a resolution of 1 mm and established that sufficient scan points would be produced at more distant sections of the vertical panels. A larger target would be more appropriate for the TLS point cloud registration, but the chosen dimensions of the target enable its easy transportation.

### 2.2. Determining the Coordinates of the Reference Target Points

When working with plane targets, the reference target point is the center of the circle. In spheres, the reference target point is the center of the sphere, whereas in cones, it is represented by the apex of the cone. In our experiment (Section 2.5), the coordinates of the reference target points were determined in all three datasets (referential measurements taken with a Leica Nova MS50 MultiStation (henceforth MS50), the UAV images or point clouds, and the TLS point cloud) and were later used to register TLS and UAV point clouds. Prior to determining the coordinates of the reference target points in the point clouds, we manually cut out the point cloud of the individual target.

#### 2.2.1. Determining the Coordinates of the Reference Point for the New Target

The reference coordinates of the new target were defined with a classic polar measurement with MS50 and the use of a miniprism in both faces.

Image matching is used to measure the coordinates of the reference target points on images in an aerial photogrammetric block: This can be performed, for instance, with least-squares matching [65]. We used Agisoft PhotoScan software, v. 1.4.1 [66], to measure the image coordinates of the reference target points. The spatial coordinates of the reference target point were calculated from the known parameters of the interior and exterior orientation of the camera, obtained in the SfM process.

**Figure 2:**
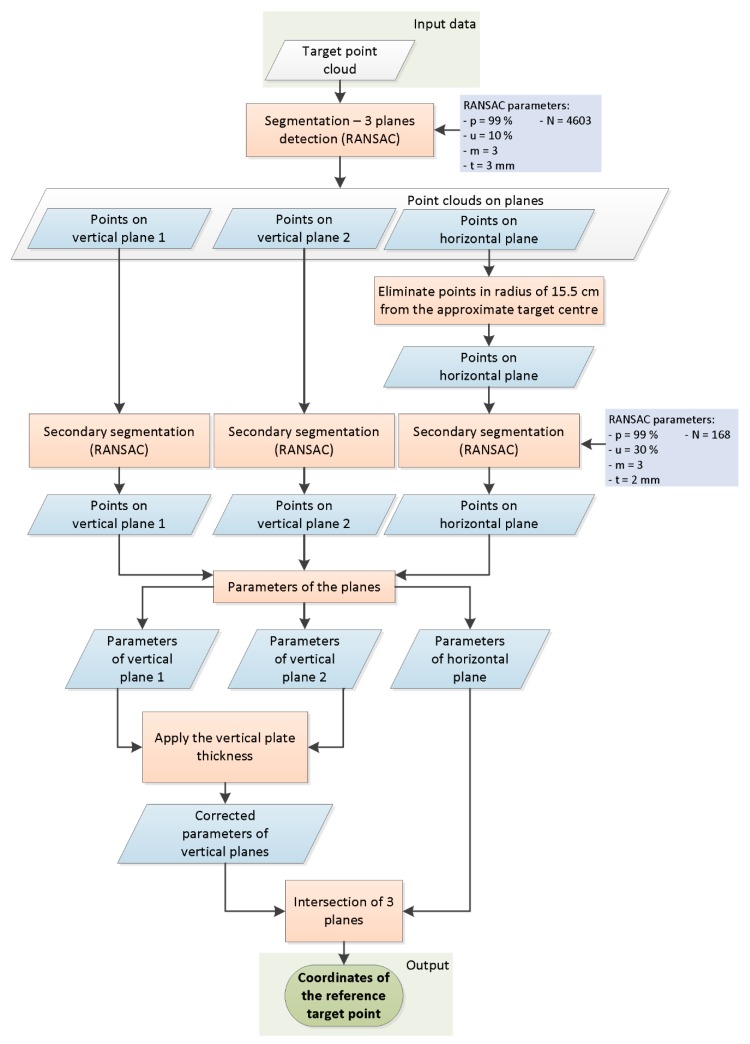
Diagram of algorithm used to define coordinates of reference point for new target.

In the TLS point cloud, the reference target point is defined by the cross-section of the three planes, one horizontal and two vertical. We created an automatic algorithm for calculating the coordinates of the reference target point. The algorithm is presented in Figure 2 and was practically realized in programs we wrote in the MATLAB environment.

**Figure 3:**
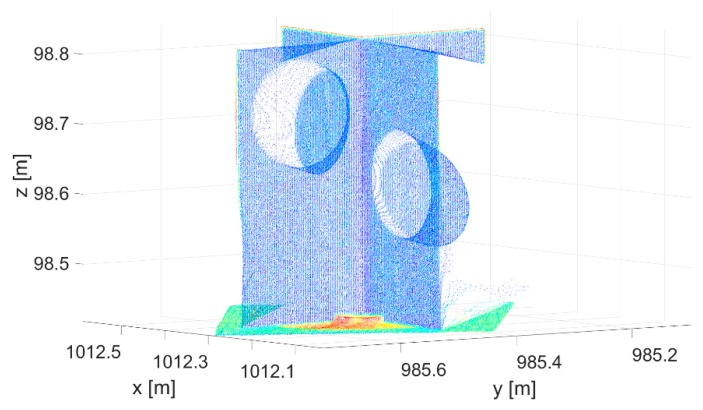
Input point cloud of the target.

**Figure 4:**
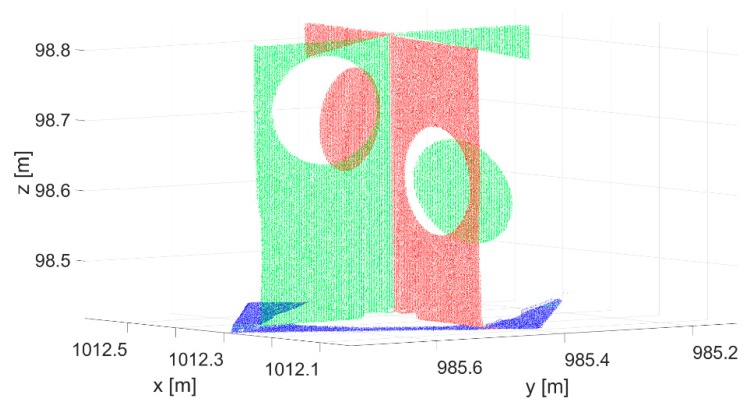
Segmented point cloud of the new target, following primary segmentation.

The input data are represented by a cut-out point cloud of the target and its vicinity (Figure 3). With the primary segmentation performed using RANSAC [67], we located the points within the input point cloud that belonged to the three planes (Figure 4). Following primary segmentation, we eliminated the systematic errors that appeared on the horizontal plane (description in the following paragraph) and performed secondary RANSAC segmentation. In order to find three different planes, RANSAC was performed repeatedly. After the first plane was segmented, the points that belonged to that plane were removed from the point cloud. The process was repeated until the third plane and its points were located. The parameters used in the process of primary and secondary RANSAC segmentation are presented in Figure 2. Parameters are denoted as follows: *p* is the probability that inliers will be randomly chosen in at least one of the iterations; *u* is the expected percentage of inliers in a point cloud; *m* is the minimum number of points that uniquely define the model; *t* is the threshold, with points within it being considered inliers; and *N* is the number of iterations.

**Figure 5:**
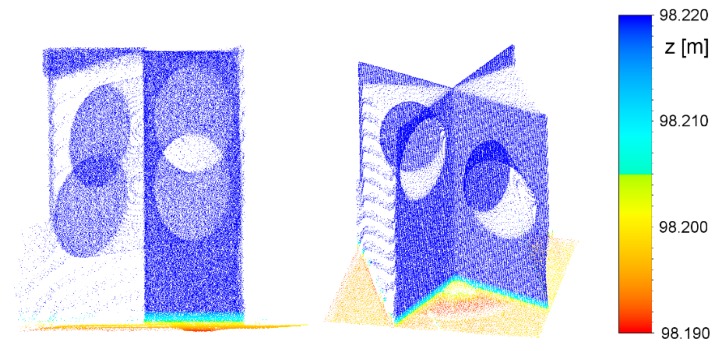
Elongated distances to the points on the black part of the horizontal plane.

Figure 4 shows that only points on the white part of the lower plane are segmented in primary RANSAC segmentation. The target’s lower panel is scanned at a large incidence angle. Several authors have studied the influence of the incidence angle and the reflectivity of a material on reflectorless TLS distance measurements. The noise level increases with higher incidence angles and range, which in turn influences the precision of measuring the distance [68]. The offset of the distance measurement is also connected to the target reflection properties [32,34] and the algorithms used to calculate the distance, which may vary depending on the type of scanner [69]. Some authors have established that materials with lower reflectivity produce more noise [70,71]. In our case, we noticed elongated distances on the black circle of the horizontal panel. The effect is shown in Figure 5 (in order to create the figure, one of the targets was rotated to a vertical position and colored by height). In order to reduce the influence of the described errors on the parameters of the horizontal plane, we excluded the points that lay within a 15.5-cm radius from the approximate position of the reference target point (the radius of the black circle is 15 cm) of the horizontal plane point cloud obtained through primary segmentation.

**Figure 6:**
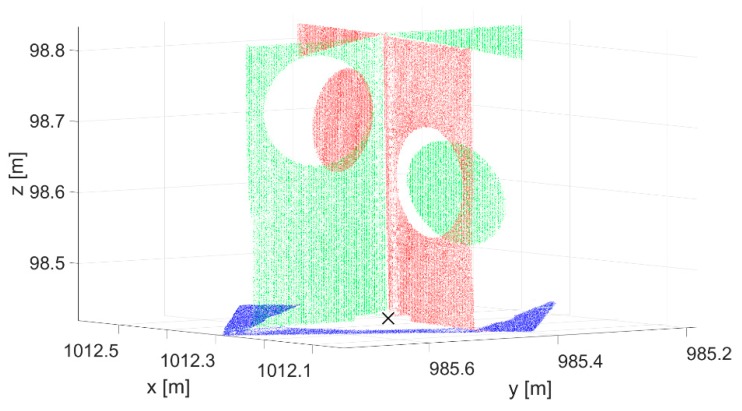
Segmented point cloud of the new target, following secondary segmentation. Reference target point is marked by “×”.

This was followed by secondary segmentation of every plane segment obtained by primary segmentation (separately), this time using a lower tolerance (2 mm) for the point distances from the plane in RANSAC. Figure 6 shows that this eliminated a few points around the intersection of the vertical planes, which were most likely burdened by the multipath.

The plane parameters are defined by minimizing the orthogonal distances of points in the plane. Due to the thickness of the panels and the manner in which the target was created, the cross-section of the vertical planes does not lie on the reference target point. In order to calculate the exact position of the reference target point, we needed to move the vertical planes by half of the thickness of the panel (1 mm) in the direction of the normal vector of the plane, away from the scanner. As a last step, the coordinates of the cross-section of the three planes were calculated.

Within the TLS data, the coordinates of the reference point of the new target were defined in the point cloud of each scanner station individually. The reason behind this can be found in the way the target is used and the procedure for defining the reference target point. As the upper part of the target is always turned toward the scanner station, the registered point cloud from all scanner stations shows a group of points on the vertical planes in various positions above the horizontal plane, which does not permit the use of the developed algorithm when determining the reference target point. The final coordinates of the reference point for the new target were calculated as the mean values, obtained from georeferenced point clouds from individual scanner stations. The precision of defining the final coordinates of the reference point of the new target is also burdened by the quality of registration of the TLS point clouds, which is why this was estimated with the use of the standard deviation [72] of the coordinates of the reference target point from *n* scanner stations.

#### 2.2.2. Determining the Coordinates of the Reference Target Points on the Remaining Used Targets

In this section, we refer to the targets that we used in our experimental study. The details of those targets are given in Section 2.5.

The coordinates of the reference point for the retroreflective target were defined through referential measurements with an MS50 and in the same way as for the new target in the UAV point cloud (Section 2.2.1). We determined the coordinates of the reference point for the retroreflective target in the TLS point cloud using the procedure described in Reference [73]. This procedure stipulates that the coordinates need to be determined by an evaluation of the precision of the coordinates by error propagation law.

The coordinates of the reference target points of all volume targets, both spheres and the cone, were defined with the least squares method, which is described in Reference [74]. The parameters of the geometric forms were obtained by solving the mathematical model. In the stochastic part of the adjustment, the covariance matrix was used to calculate the precision of all parameters. In order to calculate the coordinates and the precision of the coordinates of the reference target points, we wrote new scripts in the MATLAB environment.

### 2.3. Point Cloud Registration

The point cloud registration was performed with a Helmert transformation of the point cloud from the source coordinate system into the target coordinate system of a different point cloud. Equation (1) of the transformation is defined by 7 parameters:

- 3 translations in the directions of the coordinate axes: Δx, Δy, and Δz;
- 3 rotations around the coordinate axes: $\omega_{x}$, $\omega_{y}$, and $\omega_{z}$;
- a change in the scale: m.

Equation (1) is

(1) $$\left[\begin{array}{c} x_{trans}\\ y_{trans}\\ z_{trans} \end{array}\right]=m\cdot R\cdot \left[\begin{array}{c} x_{source}\\ y_{source}\\ z_{source} \end{array}\right]+\left[\begin{array}{c} \Delta x\\ \Delta y\\ \Delta z \end{array}\right],$$

where $\left(x_{source},\;y_{source},\;z_{source}\right)$ are the coordinates in the source coordinate system, and $\left(x_{trans},\;y_{trans},\;z_{trans}\right)$ are the transformed coordinates in the target coordinate system. The rotations around the coordinate axes form the rotation matrix *R*. The procedure to solve Equation (1) is described in Reference [43] (pp. 14–18).

We calculated the parameters of the transformation on the basis of the coordinates of the reference target points of the individual tie points (the parameters were calculated separately for each tie point type). We used the specified transformation parameters to transform the check points—signalized with the retroreflective targets—into the target coordinate system.

The following transformations from the “source” to the “target” point cloud were performed:

- Transformation of the UAV point cloud into the TLS point cloud;
- Transformation of the UAV point cloud into referential data, measured with MS50;
- Transformation of the TLS point cloud into referential data, measured with MS50.

The calculation of the transformation parameters and the data transformation were performed with the scripts we wrote in the MATLAB environment.

### 2.4. Quality of Data Registration

The quality of the registration was evaluated on the basis of the differences between the coordinates of the retroreflective check points in the target coordinate system and the transformed coordinates of the check points. As a measure of accuracy, we used the root mean square error (*RMSE*), which is often used as a measure for spatial data accuracy [75].

The coordinate differences (Δx,Δy,Δz) on the check points were calculated with Equation (2):

(2) $$\left[\begin{array}{c} \Delta x\\ \Delta y\\ \Delta z \end{array}\right]=\left[\begin{array}{c} x_{target}\\ y_{target}\\ z_{target} \end{array}\right]-\left[\begin{array}{c} x_{trans}\\ y_{trans}\\ z_{trans} \end{array}\right],$$

where $\left(x_{target},\;y_{target},\;z_{target}\right)$ are the coordinates of the check point in the target coordinate system, and $\left(x_{trans},\;y_{trans},\;z_{trans}\right)$ are the coordinates of the check point from the second dataset transformed into the target coordinate system.

Equations (3) and (4) and the coordinate differences were used to calculate the 2D and 3D positional differences Δ:

(3) $$\Delta_{2D}=\sqrt{\Delta x^{2}+\Delta y^{2}},$$

(4) $$\Delta_{3D}=\sqrt{\Delta x^{2}+\Delta y^{2}+\Delta z^{2}}.$$

The *RMSE* was calculated by coordinate axes *x*, *y*, *z* and their 2D and 3D positions with Equations (5)–(7):

(5) $$RMSEx=\sqrt{\frac{\sum_{i=1}^{n}\Delta x_{i}^{2}}{n}},\;RMSEy=\sqrt{\frac{\sum_{i=1}^{n}\Delta y_{i}^{2}}{n}},\;RMSEz=\sqrt{\frac{\sum_{i=1}^{n}\Delta z_{i}^{2}}{n}}$$

(6) $$RMSE_{2D}=\sqrt{\frac{\sum_{i=1}^{n}\Delta_{2D}{}_{i}^{2}}{n}}=\sqrt{RMSEx^{2}+RMSEy^{2}},$$

(7) $$RMSE_{3D}=\sqrt{\frac{\sum_{i=1}^{n}\Delta_{3D}{}_{i}^{2}}{n}}=\sqrt{RMSEx^{2}+RMSEy^{2}+RMSEz^{2}},$$

where n represents the number of check points.

### 2.5. Experimental Setup

**Figure 7:**
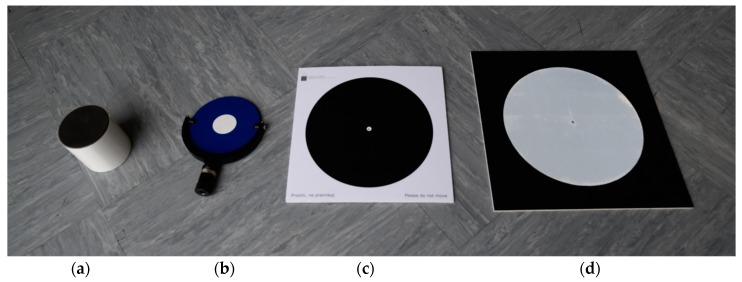
Targets for georeferencing TLS and UAV point clouds: (**a**) Cylindrical retroreflector for the registration of individual scanner stations’ point clouds; (**b**) Tilt & Turn target for georeferencing TLS point clouds; (**c**) photogrammetric ground control point (GCP); (**d**) retroreflective target that was used as a check point.

In order to analyze the appropriateness of artificial targets for the registration of TLS and UAV point clouds, we created a test area (Section 2.5.1) and scanned it with TLS and photographed it with a UAV. We placed artificial targets of various shapes and sizes throughout the test area. The first types of targets (Figure 7a–c) were used for georeferencing TLS and UAV point clouds:

- Riegl cylindrical retroreflector with a diameter of 10 cm for registering TLS point clouds from different scanner stations;
- Leica HDS 6′’ Tilt & Turn target used as a GCP for georeferencing TLS point clouds;
- Photogrammetric GCP (a black circle with a diameter of 27 cm on a white background) for georeferencing UAV aerial images.

**Figure 8:**
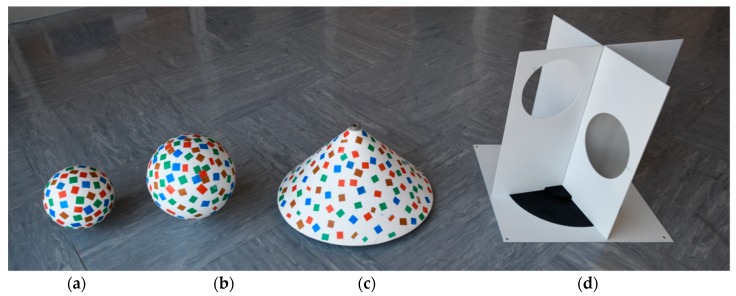
Volume targets for registering TLS and UAV point clouds: (**a**) Small sphere; (**b**) large sphere; (**c**) cone; (**d**) new target.

The second types of targets were volume targets (Figure 8), which were used as tie points for registering TLS and UAV point clouds:

- Small sphere, diameter of 15 cm;
- Large sphere, diameter of 20 cm;
- Right circular cone, diameter of 40 cm;
- New target.

The spheres and cones were painted matte white, and then various colored stickers were attached to the bodies in a random pattern. This was performed so that the featured points could be better identified with the SfM algorithms.

We evaluated the quality of registering the point clouds on check points made from retroreflective targets, which were created especially for this study (Figure 7d). The target is in the form of a black plane, 40 cm × 40 cm × 0.5 cm, in the center of which a circle made from retroreflective foil, measuring 30 cm in diameter, is stuck. The retroreflective target enables precise measurements in TLS and UAV point clouds.

The precise measurements of all tie point targets, GCPs, and check points were performed with an MS50, which enables laser scanning and also classic geodetic measurements.

#### 2.5.1. Field Measurements and Initial Data Processing

**Figure 9:**
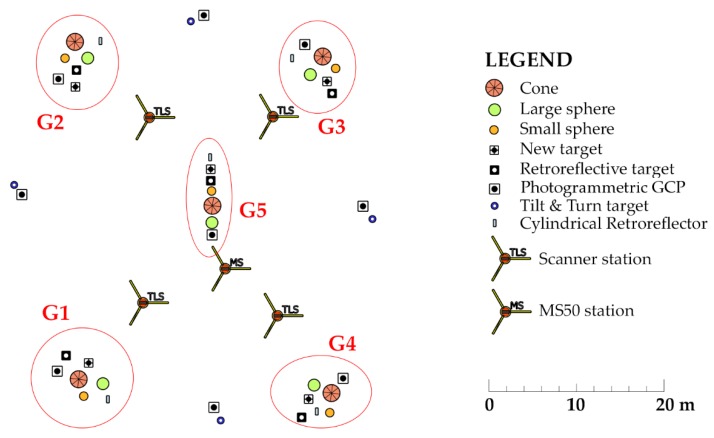
Distribution of targets, TLS scanner stations, and MS50 station throughout the test area.

Within the test area, which measures approximately 55 m × 55 m, we stabilized GCPs, check points, and tie point targets in five groups (Figure 9 shows groups G1–G5, marked by ellipses). We analyzed their appropriateness for registration of the point clouds in our research. Each group included the following targets:

- Tie points (small and large sphere, cone, and new target);
- Photogrammetric GCP;
- Cylindrical retroreflector for registering TLS point clouds;
- Retroreflective check point target.

The groups were organized throughout the test area so that one group of targets was in every corner of the test area and one was in the center of the area (Figure 9). Apart from the targets that were stabilized within the groups, an additional 4 photogrammetric GCPs for georeferencing the UAV point cloud and 4 Tilt & Turn targets for georeferencing the TLS point cloud were stabilized outside of the groups.

**Figure 10:**
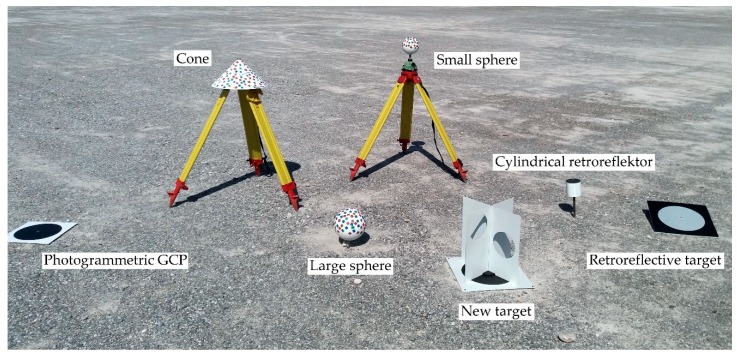
Group of targets in the test area.

Target stabilization within a group is shown in Figure 10. The retroreflective check point targets were raised by approximately 10 cm on the outer side of the area. This reduced the incidence angle of the TLS measurement rays and thus increased the quality and number of measured points on the target without deforming the shape of the circle on the aerial images.

#### 2.5.2. Referential Measurements

We used the MS50 measurements to define the local coordinate system, which we treated as the reference coordinate system. The coordinates of the centers of the plane targets (new targets, retroreflective check point targets, photogrammetric GCPs, and Tilt & Turn targets) were defined with the classic polar measurement method on both faces by signalizing the centers of the targets with a miniprism or, in the case of the Tilt & Turn target, with direct sighting of the center of the target. When we were determining the coordinates, we took into account the meteorological parameters (temperature and air pressure), which we measured with a Meteo Station HM30, and entered the data into the MS50. The volume targets (small spheres, large spheres, and cones) were scanned at a resolution of 2.5 mm × 2.5 mm. The MS50 enables measurements on the prism (single measurement) with an angle and distance accuracy of 1” and 1 mm + 15 ppm, respectively. The declared accuracy and precision of scanning in the 1000-Hz mode is 1 mm at 50 m.

#### 2.5.3. Terrestrial Laser Scanning and Processing of the TLS Point Cloud

The laser scanning was performed with a Riegl terrestrial laser scanner VZ-400, which was controlled with the RiSCAN PRO software. The scanner has a declared scanning precision and accuracy of 3 mm and 5 mm, respectively. We performed the scanning with 4 stations (Figure 9) positioned throughout the test area. We created an overview scan on every scanner station and a detailed scan of the tie points and retroreflective check points with a resolution of 1 mm × 1 mm on the target. The software has its own way of scanning TLS tie targets (cylindrical retroreflector and flat Tilt & Turn target) and a built-in algorithm, with which it calculates the coordinates of the tie points in the coordinate system of an individual scanner station. Based on the coordinates of the cylindrical retroreflectors, we registered all four TLS scans with RiSCAN PRO software. The registered TLS point cloud was georeferenced into the reference coordinate system with the coordinates of the Tilt & Turn targets, which were measured with the MS50. The registration precision of the scanner stations is shown with the standard deviation of the registered coordinates of the tie points (Table 1). The TLS point cloud georeferencing precision was 3.6 mm.

#### 2.5.4. Photogrammetric Surveying and the Creation of UAV Point Clouds

A UAV DJI Phantom 4 PRO with an FC6310 camera with a focal length of 8.8 mm was used for photogrammetric surveying. In the UAV point cloud, the number of points on an individual target depended on the size of the target and the altitude of the flight, which at the given focal length and size of the image sensor determined the ground sample distance (GSD) of the aerial images. In order to check the suitability of the individual artificial targets at various GSDs, we performed three surveys with the UAV at various altitudes, which gave us the following approximate average GSDs of aerial images: 0.5, 1.0, and 2.0 cm. Only nadir images were taken in all three surveys.

**Figure 11:**
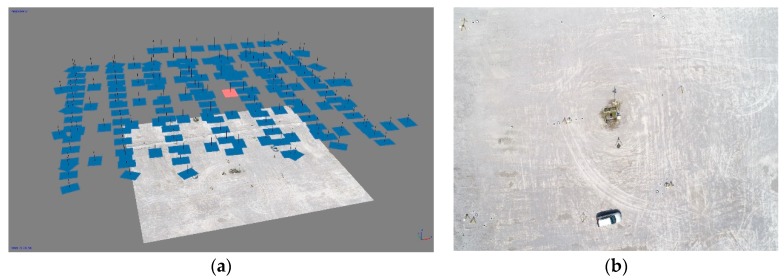
Photogrammetric survey: (**a**) Photogrammetric block UAV40 (oriented images and dense point cloud); (**b**) image sample (colored in red in (**a**)).

We used the obtained images to create three photogrammetric image blocks (UAV20, UAV40, and UAV75), which were in turn used to create UAV point clouds. The images were recorded with an 80% longitudinal and 70% transversal image overlap. The photogrammetric block UAV20 is comprised of images recorded in one flight direction, at an altitude of 20 m above ground level. The blocks UAV40 (Figure 11) and UAV70 comprise images recorded from two perpendicular flight directions, which differ in their altitudes by between 6 and 8 m. This improved the inner geometry of the block of aerial photographs in bundle block adjustment, which in turn improved the vertical accuracy of the model [76]. The recording characteristics of the individual block are presented in Table 2.

**Figure 12:**
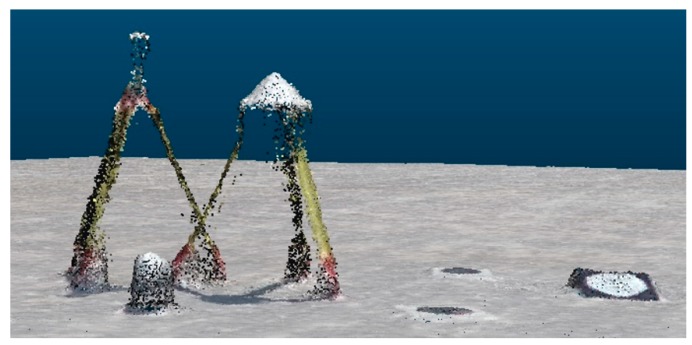
Segment taken from a dense point cloud, created from a UAV40 photogrammetric block.

The individual blocks were photogrammetrically processed with PhotoScan, which uses SfM to create dense point clouds (Figure 12). The external orientation of the images and the georeferenced dense point clouds were calculated with the coordinates of the photogrammetric GCPs measured by the MS50. The quality of the georeferencing was assessed with the check points, i.e., retroreflective targets and new targets. The accuracy of the georeferencing UAV point clouds is shown in Table 3, in which the *RMSE* is given in cm. The results show that the $RMSE_{2D}$ on the check points is within 1 cm for all missions, whereas the $RMSE_{3D}$ is within 2 cm, which we believe to be a good result. However, what we can also see is that the *RMSE* does not necessarily increase with a higher flight altitude. The reason for this is that the *RMSE* value depends on a number of input factors (terrain morphology, camera calibration, number and distribution of GCPs, image block composition, etc.) and not only on the flight altitude. This issue was thoroughly investigated and explained in Reference [77].

## 3. Results

Section 3.1 presents the precision of defining the coordinates of the reference target points of the artificial targets, which we used as tie points for registering TLS and UAV point clouds or as check points for evaluating the performed registration. Section 3.2 describes the quality of registration of the UAV and TLS point clouds, taking into account the types of tie points used.

### 3.1. Precision of Coordinates of Reference Target Points

Table 4 and Table 5 provide 2D and 3D precisions, respectively, of the coordinates of the reference target points for the referential measurements with MS50 for the TLS point cloud and three UAV point clouds (UAV20, UAV40, UAV75). The precision of the coordinates in the tables is defined by the standard deviation, provided in cm. The exceptions are the accuracies of the coordinates of the reference points for the new and retroreflective targets in the UAV point clouds. These are provided in cm with *RMSE*, calculated from the differences between the coordinates of the referential measurements with MS50 and the coordinates of these points, estimated by aerial triangulation. In Table 4 and Table 5, we marked tie points with the following abbreviations: “sSph” for small sphere, “LSph” for large sphere, and “Cone” and “newT” for the new target. The retroreflective check point target is marked by the abbreviation “rRef”.

As expected, Table 4 and Table 5 show that the reference target points were defined with the greatest precision for the dataset of the referential measurements with MS50. Similar precision for the coordinates of the reference points for volume targets was achieved in the TLS dataset. Somewhat poorer, in the range of just under 5 mm, were the reference points of the new and retroreflective targets defined in the TLS dataset. When compared to the precision of the coordinates of the remaining reference target points, the coordinates of the centers of the spheres were much more precisely defined in all datasets. The reason behind this lay in the use of RANSAC for the segmentation of the point cloud of the sphere points, where, by selecting the tolerance value, we eliminated the blunders. The estimated precision of the calculated coordinates of the centers of the spheres was thus overrated. The centers of the large spheres were defined with the greatest precision in all UAV point clouds, regardless of the spatial resolution. The importance of the spatial resolution of the images is shown in the poor definition of the reference target points in aerial photogrammetric datasets. At a GSD of 1 cm and 2 cm (UAV40 and UAV75), the worst defined were the apexes of the cones, whereas at a GSD of 0.5 cm (UAV20), the plane targets were the worst defined. Roughly speaking, we could say that the volume targets were more precisely defined than the plane targets when the highest spatial resolution was used, whereas at a lower spatial resolution the situation was reversed (if we excluded the sphere targets, whose estimated precision was overrated). In the UAV75 point cloud produced from images with a spatial resolution of 2 cm, the small sphere with a diameter of 15 cm failed to provide an appropriate number of quality points to determine the center of the target. Three of the five targets revealed merely 4 or 7 points.

### 3.2. Quality of Registration

The quality of registering TLS and UAV point clouds was estimated on the basis of the *RMSE* of check points, signaled with a retroreflective target. As a comparison, Section 3.2.1 presents the *RMSE* prior to the performed registrations with the analyzed tie points.

#### 3.2.1. Discrepancies of TLS and UAV Point Clouds on the Check Points Prior to Registration

**Figure 13:**
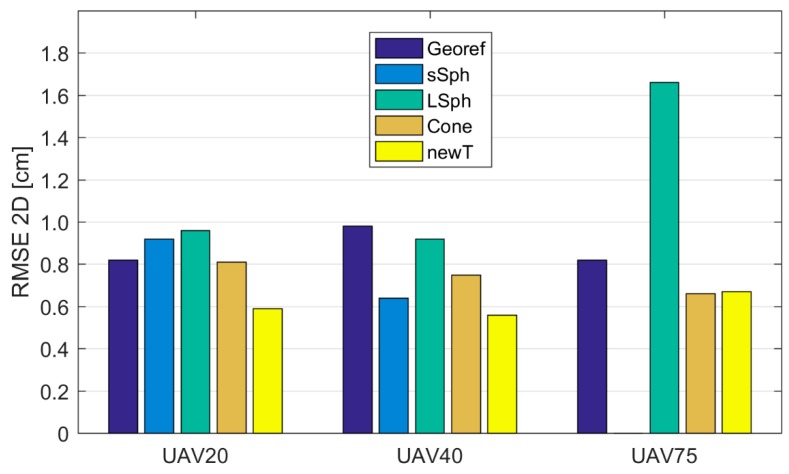
The $RMSE_{2D}$ on check points, following the registration of UAV and TLS point clouds, taking into account the used tie point, given in cm. “Georef” represents the *RMSE* prior to the registration of the TLS and UAV point clouds.

**Figure 14:**
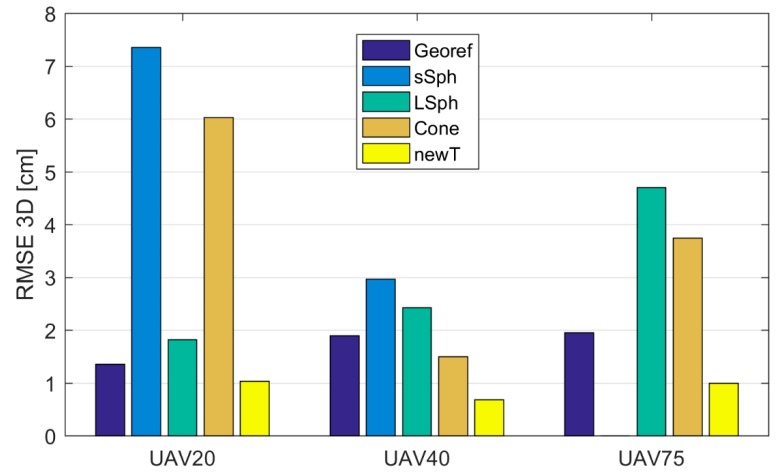
The $RMSE_{3D}$ on check points, following the registration of UAV and TLS point clouds, taking into account the used tie point, given in cm. “Georef” represents the *RMSE* prior to the registration of the TLS and UAV point clouds.

The UAV point clouds were georeferenced into the reference coordinate system on the basis of photogrammetric GCPs. The TLS point cloud was georeferenced with the Tilt & Turn targets. Both types of georeferencing points were measured with the MS50. Table 6 shows the *RMSE* calculated from the coordinate differences of the retroreflective check points, defined in UAV and TLS point clouds. Figure 13 and Figure 14 show *RMSE*s calculated prior to the registration of TLS and UAV point clouds with the marking “Georef”.

#### 3.2.2. Discrepancies of the TLS and UAV Point Clouds on the Check Points after the Registration

All UAV point clouds (source coordinate system) were transformed into the TLS point cloud (target coordinate system) as described in Section 2.3. In order to define the transformation parameters, a different type of tie point was used each time. We performed 12 transformations, the qualities of which are represented by $RMSE_{2D}$ and $RMSE_{3D}$ in Table 7 and Table 8, respectively. The graphic representations of $RMSE_{2D}$ and $RMSE_{3D}$ are shown in Figure 13 and Figure 14, respectively. In Table 7, Table 8, Table 9 and Table 10, tie points are marked with the following abbreviations: “sSph” for small sphere, “LSph” for large sphere, and “Cone” and “newT” for the new target. The retroreflective check point target is marked by the abbreviation “rRef”.

Figure 14 shows that the best 3D accuracy in all UAV point clouds was achieved with the use of the new target. When using aerial datasets with spatial resolutions between 0.5 cm and 2 cm, the 3D accuracy of the new target was within 1 cm. The remaining volume targets all provided a 1-cm spatial accuracy $\left(RMSE_{2D}\right)$ when using aerial images with a GSD of at least 1 cm (UAV20 and UAV40; see Figure 13). The $RMSE_{3D}$ shows that when volume targets were used, the registration by height tended to be problematic. The worst performance was delivered by the small sphere, which was expected due to its size. Poorer quality 3D registration of the TLS and UAV point clouds was also delivered when the cone was used as a tie point.

When the obtained accuracies are compared to the accuracies from previous georeferencing attempts (Table 6), it is clear that the registrations with all types of analyzed targets at all GSDs of aerial images provided a worse or comparable accuracy, with the exception of the new target, which provided higher accuracy with all GSDs of aerial images.

**Figure 15:**
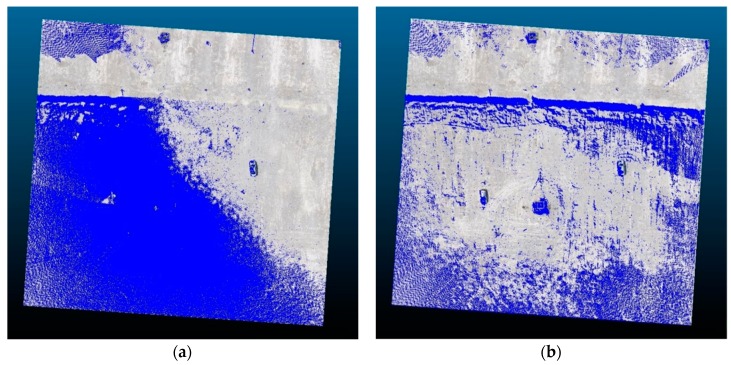
The TLS (blue color) and UAV40 (grayscale) point clouds: (**a**) Before registration; (**b**) after registration with the new target.

The suitability of the new target for registering TLS and UAV point clouds is graphically presented in Figure 15, which shows the overlap of the TLS and UAV40 point clouds once georeferencing was performed (prior to registration, see Figure 15a) and after the transformation of the UAV40 point cloud into the TLS point cloud with the use of the new target (Figure 15b). Figure 15 shows that georeferencing with the use of GCPs, measured in the same coordinate system, did not ensure appropriate alignment of the point clouds obtained with different types of technology, even when the GCPs were defined with great precision. However, we successfully aligned TLS and UAV point clouds with the use of the new target as a tie point.

#### 3.2.3. Registration of TLS and UAV Point Clouds on Referential Measurements

**Figure 16:**
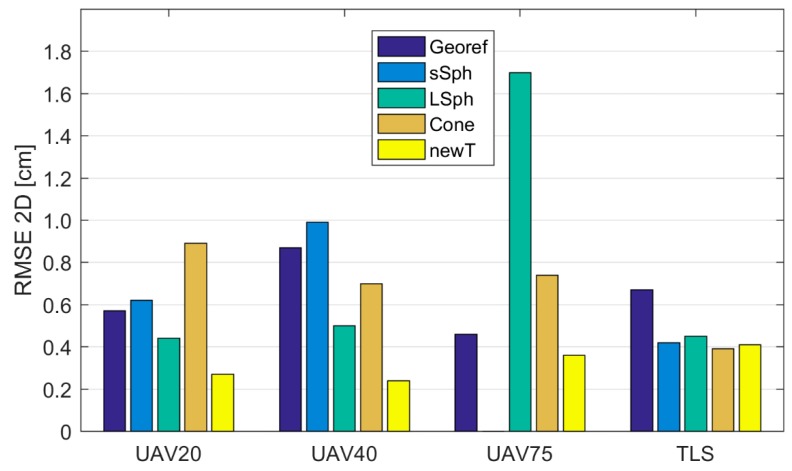
The $RMSE_{2D}$ of the check points following the UAV and TLS point cloud registration into the referential measurements performed with MS50.

**Figure 17:**
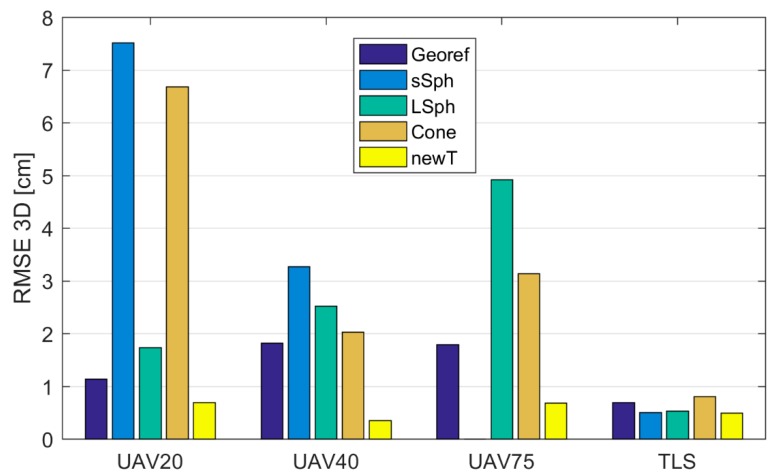
The $RMSE_{3D}$ of the check points following the UAV and TLS point cloud registration into the referential measurements performed with MS50.

The coordinates of the retroreflective check point targets and the new targets were better defined in the dataset of the referential measurements with MS50 than they were in the TLS dataset (Table 4 and Table 5). With the transformation of the UAV and TLS point clouds into referential measurements (target coordinate system), performed with MS50, we checked whether better results of registration could be obtained as a result of the higher precision of the coordinates of the reference target points in the referential measurements data. We performed 16 registrations. In order to define the transformation parameters, a different type of tie point was used each time. The quality of the registrations is represented by $RMSE_{2D}$ and $RMSE_{3D}$ in Table 9 and Table 10, respectively. The graphic representations of $RMSE_{2D}$ and $RMSE_{3D}$ are shown in Figure 16 and Figure 17, respectively. We calculated the *RMSE* from the coordinate differences of the retroreflective check point targets (measured with MS50) and the transformed coordinates of the retroreflective targets in TLS and UAV point clouds. In Figure 16 and Figure 17, *RMSE*s calculated prior to the registration of TLS and UAV point clouds (Table 6) are shown with the marking “Georef”.

Once again, the new target yielded the best results in all datasets when registering UAV point clouds with referential measurements. With the use of the new target, the $RMSE_{3D}$ of the check points was below 1 cm, while the $RMSE_{2D}$ ranged between 0.24 and 0.36 cm. The greatest discrepancies on the check points could be found in the results obtained with the small sphere. $RMSE_{3D}$, when registering with the large sphere, ranged between 1.73 cm and 4.92 cm, and when the cone was used, between 2.03 and 6.68 cm. Within the UAV point clouds for all volume targets (spheres and cones), defining the *z* coordinate was the most problematic, which resulted in a much higher $RMSE_{3D}$ compared to $RMSE_{2D}.$ When used with UAV point clouds, the new target was the only target, which in all cases yielded better results compared to the results of prior georeferencing. The new target yielded the most accurate results ($RMSE_{3D}=$ 0.49 cm) when registering the TLS point cloud with referential measurements. Similar accuracy was achieved with the small and large spheres.

## 4. Discussion and Conclusions

This article analyzes the use of various artificial targets as tie points for registering TLS and UAV image point clouds. We used a small and large sphere, a cone, and a new “three-plane target” created especially for this study. The analysis was performed on data obtained by terrain measurements with a terrestrial laser scanner and UAV imagery. Surveys using UAV were performed at three different flight altitudes, so that we could define the use of various tie points in relation to the GSD of the images, from which the UAV point clouds were created.

We calculated the coordinates of the reference target points in both datasets for each tie point and estimated their accuracy. For our new target, we developed a new procedure for defining the coordinates of the reference target point in the TLS point cloud. The point clouds were registered with the use of seven-parameter transformation. The transformation parameters were calculated from the coordinates of the reference target points in TLS and UAV point clouds. We performed numerous registrations of TLS and UAV point clouds for every type of tie point individually. The quality of the performed registrations was estimated using the RMSE of the check points.

**Figure 18:**
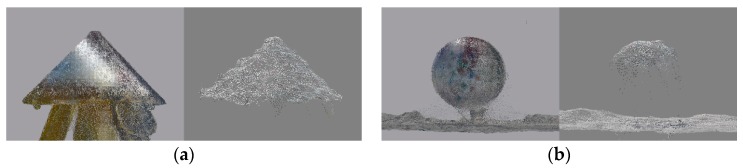
(**a**) Cone in the TLS point cloud (left) and noise-distorted cone in the UAV point cloud (right); (**b**) large sphere in the TLS point cloud (left) and flattened large sphere in the UAV point cloud (right).

Artificial targets are useful as tie points for registering two point clouds when both point clouds enable precise definition of the coordinates of the reference target point. When point clouds from the same source are merged, we can usually ensure that the accuracy and precision of the coordinates of the tie points in both point clouds are similar. When registering point clouds from different sources, as was the case in our example, the accuracy and the precision of the coordinates of the tie points differ. The precision of the coordinates of all analyzed tie points in the TLS point cloud ranged between 1 mm and 4.5 mm, whereas in the UAV point cloud it ranged between 1 mm and 52 mm (Table 4 and Table 5). We can say that the coordinates of the tie points in UAV point clouds were roughly 10 times less precise. The worst precision was calculated for the *z* coordinate, which has also been ascertained by other authors [21,23,59]. Through a visual overview of the UAV point cloud, which we have not described yet in this article, we ascertained that the volume targets (spheres and cones) yielded a lot of noise, which distorted the geometry of the targets. Most likely, the reason for this lay in overexposed aerial images, as the white volume targets were brighter than their surroundings. Consequently, the volume targets in the created UAV point clouds were slightly flattened (Figure 18). A critical review and analysis of selected dense image-matching algorithms was presented by Remondino et al. [3]. The datasets they adopted for testing included terrestrial and aerial image blocks acquired with convergent and normal (parallel axes) images at different scales and resolution. The authors reported several reasons for problems in dense matching algorithms. These reasons were initial image quality (noise, low radiometric quality, shadows, etc.); poor image configuration; certain surface materials (shiny or textureless objects); or scene/object characteristics, such as the presence of shadows, sharp discontinuities, and small structures. This could result in noisy point clouds, smoothing effects, and/or difficulties in feature extraction. The fact that varying the illumination of images also affects image tie point matching was reported by Gerke, Nex, and Jende [6] in their representation of ISPRS benchmark results of multiplatform photogrammetry.

The precision of the coordinates of the reference target points in the UAV point cloud was dependent on the GSD of the images (Table 5) and their photographic quality, which directly influenced the geometrical quality of the created UAV point cloud. The image GSD could be improved with a lower flight altitude, which extends the time for terrain measurement and the processing of images with SfM [19]. The second possibility for improving the point cloud of the volume tie points is to use larger targets. By increasing the size of the targets, we would get more points on the target and the possibility of using the targets from greater flight altitudes. The downside of the larger-volume targets is that they are harder to transport and are less flexible to work with.

Using a sphere target measuring 20 cm in diameter, we could register the TLS point cloud and the UAV point cloud made from images with a GSD of 1 cm or less, with an accuracy of approximately 2.5 cm (Table 8). The small sphere, measuring 15 cm in diameter, yielded approximately three times worse accuracy. When a UAV point cloud is created from images with a GSD of approximately 1 cm, we suggest that a sphere target with a diameter of at least 20 cm be used for the registration of the TLS and UAV point clouds. The cone-shaped target yielded poorer results when registering point clouds UAV20 and UAV75 with the TLS point cloud ($RMSE_{3D}$ ranging from 3.7 cm to 6 cm). However, it provided better results when registering the UAV40 point cloud (GSD of the images ≈1cm), where the accuracy was estimated with an $RMSE_{3D}$ of 1.5 cm.

The new target yielded the best 3D accuracy as a tie point for all UAV point clouds (GSD of the images ranging from 0.5 cm to 2 cm). The $RMSE_{3D}$ did not surpass 1.1 cm. The poorer quality of the UAV point clouds on the tie points did not influence the precision of the coordinates of the reference point for the new target. The reason for this lay in the way the coordinates are defined in the UAV dataset. They are calculated with the use of SfM from the image coordinates, which are obtained automatically with least squares matching. In our case, the image measurements enabled a more precise calculation of the coordinates of the reference target point than the modeling of the UAV point clouds did. With the latter, we calculated the reference target points for volume targets. In the TLS point cloud, the reference point of the new target is defined by the cross-section of the three planes, which coincides with the center of the black circle, which represents the reference target point in the UAV dataset. When compared to the volume targets, the precision of the coordinates of the reference target point in the TLS point cloud was slightly lower, but still within a few millimeters. The geometrical relation between the scanner and the target represents the main influence, as the incidence angle of the laser beam on the horizontal plane of the target can be very high when measuring from long distances. The achieved results confirm the good design of the target and its suitability for registering TLS and UAV point clouds whenever a high-quality registration is needed.

By analyzing the registration results, one can see that the *RMSE* of the registered point clouds was lower than the measurement precision of the new target. To assess the quality of the new target position, its 2D and 3D precision were calculated in all datasets. The new target was provided by the reference coordinates by measuring with the MS50 in both faces using a miniprism. The precision of the reference coordinates was assessed, taking into account the accuracy of the MS50 and using error propagation law. In this case, the new target coordinates were determined with an $RMSE_{3D}$ of 1.7 mm (Table 5). In the TLS point clouds, the new target positions were determined in each scan station individually. Due to the new target geometry, which allows for the rotation of vertical panels with holes toward the scanner, the coordinates of the new target were defined as the arithmetic mean of the quadruple-calculated coordinates. This was also related to the procedure for determining the precision of a new target in the TLS point cloud. We calculated it as a standard deviation of four-times-determined coordinates. The $RMSE_{3D}$ of the new target in the TLS point clouds was 4.3 mm (Table 5) and was related to the quality of the scan stations’ registration with Riegl cylindrical retroreflectors. The positions of new targets and their *RMSE*s in the UAV point cloud were determined using the automatic procedure in PhotoScan. The precisions were between 11.5 mm and 20.3 mm (Table 5) for three different flight altitudes. With respect to the measurement equipment, the precision of the new target positions in the various datasets was within the expected values. When we used the new target to register the UAV and TLS point cloud at the check points, we received an $RMSE_{3D}$ less than 7 mm in all registrations (Table 10). The reason for the $RMSE_{3D}$ being up to three times lower at check points than the $RMSE_{3D}$ of the new target positions in the UAV point clouds was the calculation mode. The $RMSE_{3D}$ was calculated from the differences in the positions of the check points in the target coordinate system after the transformation. The precision of the new target was therefore not included in the calculation of the quality of the registration.

One of the main shortcomings of the new target is that it cannot be used from further distances from the scanner. In that case, the incidence angles are too large, which affects the measurement accuracy of the horizontal plane of the new target. The incidence angle and the range affect the individual point signal-to-noise ratio, and a larger standard deviation of errors in the direction of the laser beam was observed for larger incidence angles in Reference [68]. Lichti [78] suggests using the a priori threshold of a maximum incidence angle of 65° for removing nonreliable measurements. Therefore, careful planning for TLS measurements is essential when using the new target. The targets can be set on the border of the scanning area and are preferably tilted toward the scan stations to improve the scanning geometry. Future work will be performed to model the range and incidence noise with the methods described in Reference [68]. Even if we can model the noise, we must prevent the horizontal plates of the targets from being perpendicular to the incident rays of the scanner, which is sometimes difficult to control in practical settings. In these cases, the calculation of the reference target point’s coordinates with a cross-section of three planes will not be possible because we will have no points for modeling the horizontal plane of the target.

A systematic error resulting from the new target design is the generation of mixed pixels [79] that appeared in the TLS point cloud, which are clearly visible in Figure 5. This data artifact was caused due to nonzero laser beam width. A pulsed laser scanner without full-waveform recording capability cannot discriminate between the returns from two surfaces separated by less than half the pulse length, so point measurements appeared in the range discontinuity region between the two surfaces [27]. Segmentation of the target planes using RANSAC removed all mixed pixels (Figure 4 and Figure 6). Nevertheless, the influence of the mixed pixels on the reference target point determination should be investigated in future work.

In our experiment, the new target and volume targets were manually cropped from the point clouds before determining the coordinates of the reference target points. This task could possibly be automatized for the new target, as in our case we first used GCPs to coarsely register the point clouds. As the image coordinates of the new targets were automatically measured with PhotoScan, and their 3D reference coordinates were calculated in SfM, we could use these coordinates to obtain conjugate point clouds of the new target in the TLS point cloud. However, due to the small dimensions of the used targets, we found it difficult to search them automatically in the point clouds. In the case of point clouds not being aligned, the automation of the registration process would be a demanding task, a challenge to be addressed and solved in the future.

By analyzing the available data, we ascertained that the image GSD had a relatively small influence on the accuracy of the registration of TLS and UAV point clouds, ranging between 0.5 and 2 cm. In contrast, the type of target that we used as a tie point influenced the quality of registration. The new target provided the highest accuracy in all the analyzed examples. It should be mentioned that better photographic quality of aerial images might yield higher-quality results when working with volume targets. Due to the various restrictions of projects, it is impossible to freely choose the time for terrain measurements in practice, which means that we do not always have optimal weather and light conditions. The new target proved to be the most optimal tie point for the registration of TLS and UAV point clouds in poorer conditions as well. We can also use it as the GCP for georeferencing the point cloud into the desired coordinate system.

## Figures and Tables

**Table 1:** Standard deviation of the registration on TLS scanner stations.

Scanner Station	σ_3D_ (mm)
S1	1.0
S2	1.3
S3	1.7
S4	2.2

**Table 2:** Characteristics of the photogrammetric blocks. GSD: Ground sample distance.

Block	Flight Altitude (m)	Number of Images	GSD (cm)
UAV20	20	112	0.49
UAV40	Transversal 40, longitudinal 46	175	1.09
UAV70	Transversal 75, longitudinal 83	50	2.06

**Table 3:** Root mean square error (*RMSE*) on GCPs and check points after the georeferencing of the photogrammetric image blocks. The *RMSE* is given in cm.

Point Type	$\mathrm{UAV}20\;RMSE_{2D}$	$\mathrm{UAV}20\;RMSE_{3D}$	$\mathrm{UAV}40\;RMSE_{2D}$	$\mathrm{UAV}40\;RMSE_{3D}$	$\mathrm{UAV}75\;RMSE_{2D}$	$\mathrm{UAV}75\;RMSE_{3D}$
GCP	0.58	1.03	0.87	2.01	0.39	1.38
check	0.54	1.14	0.85	1.93	0.43	1.77

**Table 4:** Two-dimensional precision of coordinates of reference target points (in cm). sSph: Small sphere; LSph: Large sphere; new T: New target; rRef: Retroflective check point target.

Point Type	MS50	TLS	UAV20	UAV40	UAV75
sSph	0.004	0.004	0.22	0.71	/
LSph	0.003	0.003	0.07	0.21	0.45
Cone	0.02	0.01	0.15	0.39	0.70
newT	0.17	0.29	0.50	0.84	0.39
rRef	0.15	0.38	0.57	0.87	0.46

**Table 5:** Three-dimensional precision of coordinates of reference target points (in cm).

Point Type	MS50	TLS	UAV20	UAV40	UAV75
sSph	0.005	0.005	0.24	0.76	/
LSph	0.004	0.003	0.08	0.26	0.55
Cone	0.12	0.14	0.65	2.40	5.23
newT	0.17	0.43	1.15	2.03	1.76
rRef	0.15	0.45	1.14	1.82	1.79

**Table 6:** Root mean square error (*RMSE*) calculated from differences between coordinates of the check points in TLS and UAV point clouds, prior to the performed registration.

*RMSE* (cm)	UAV20	UAV40	UAV75
2D	0.82	0.98	0.82
3D	1.36	1.90	1.94

**Table 7:** The $RMSE_{2D}$ on check points, following the registration of UAV and TLS point clouds, taking into account the used tie point (in cm).

Tie Point	UAV20	UAV40	UAV75
sSph	0.92	0.64	/
LSph	0.96	0.92	1.66
Cone	0.81	0.75	0.66
newt	0.59	0.56	0.67

**Table 8:** The $RMSE_{3D}$ on check points, following the registration of UAV and TLS point clouds, taking into account the used tie point (in cm).

Tie Point	UAV20	UAV40	UAV75
sSph	7.36	2.97	/
LSph	1.82	2.43	4.70
Cone	6.03	1.50	3.74
newt	1.03	0.68	1.00

**Table 9:** The $RMSE_{2D}$ of the check points following the UAV and TLS point cloud registration into the referential measurements performed with MS50, provided in cm.

Tie Point	UAV20	UAV40	UAV75	TLS
sSph	0.62	0.99	/	0.42
LSph	0.44	0.50	1.70	0.45
Cone	0.89	0.70	0.74	0.39
newT	0.27	0.24	0.36	0.41

**Table 10:** The $RMSE_{3D}$ of the check points following the UAV and TLS point cloud registration into the referential measurements performed with MS50, provided in cm.

Tie Point	UAV20	UAV40	UAV75	TLS
sSph	7.52	3.27	/	0.50
LSph	1.73	2.52	4.92	0.53
Cone	6.68	2.03	3.14	0.81
newT	0.69	0.36	0.68	0.49
